# Essential Oil and Smoke Components of *Artemisia absinthium* and *Hagenia abyssinica*

**DOI:** 10.1155/2024/9949040

**Published:** 2024-05-23

**Authors:** Nigussie Kebie, Melaku Assefa Sisay

**Affiliations:** Department of Chemistry College of Natural and Computational Sciences Wollo University P.O. Box 1145, Dessie, Ethiopia

## Abstract

*Hagenia abyssinica* and *Artemisia absinthium* are widely distributed tree species in Ethiopia and known for their traditional medicinal uses. The present study was conducted to determine the essential oil and smoke constituents of *H. abyssinica* and *A. absinthium* leaves using GC-MS. The main components of the *A. absinthium* essential oil were valencene (5.48%), bornyl acetate (5.15%), and *trans*-cinnamic acid (4.34%). 2-Bornanone (18.54%), *o*-cymene (12.80%), and nerolidol (7.04%) were the dominant components of the MeOH fraction of the smoke derived from the leaves of *A. absinthium*, while 2-propenoic acid butyl ester (46.49%), heptadecane (10.66%), and 9-octylheptadecane (7.78%) were the major components of the *n*-hexane fraction. The main components of the *H. abyssinica* essential oil were *cis*-davanone (14.73%), Aristolediene (9.31%), and cryptone (6.50%). *β*-Myrcene (12.59%), neophytadiene (11.54%), and limonene (11.27%) were the dominant components of the MeOH fraction of the smoke derived from the leaves of *H. abyssinica*. 1,3,5,7-Cyclooctatetraene (33.58%), prehnitene (7.01%), and heptadecane (5.46%) were the dominant components of the *n*-hexane fraction of the smoke derived from the leaves of *H. abyssinica*. The smoke components of *A. absinthium* and *H. abyssinica* were reported here for the first time.

## 1. Introduction

*Artemisia absinthium* locally known as “ariti” is a perennial odorous herb which is widespread in Ethiopia. *A. absinthium* exhibited antispasmodic and antioxidant properties. It is used for treatment of cough and malaria [1, 2]. Different natural products were isolated from the roots of *A. absinthium* [3]. The chemical composition and biological activity of the essential oil from this plant were also reported several times [4–8].

*Hagenia abyssinica* is commonly known as *kosso* in Ethiopia. *H. abyssinica* have long been used to expel tapeworm, a very common infestation among Ethiopians [9, 10]. *H. abyssinica* is used in treatment of high blood pressure, fever/cough, intestinal worms (tape worm), stomachache, diarrhea, healing of wound, typhoid, cold (bronchitis), epilepsy, livestock disease (thin/skinny body), evil eye, hepatitis, sexually transmitted diseases (STDs), throat disease, problems related to bile cancer (mixed with other plants), malaria, and diabetes [11]. Several secondary metabolites were reported from the essential oil and solvent extracts of *H. abyssinica* [12–14].

Although *A. absinthium* and *H. abyssinica* are widely used for treatment of different diseases and as incense plants in Ethiopia, the chemical composition of the smoke from these plants was not reported before. This study focuses on the extraction of essential oil from the leaves of *A. absinthium* and *H. abyssinica* using hydrodistillation and collection of medicinal smoke from the leaves of *A. absinthium* and *H. abyssinica* using *n*-hexane and methanol and comparison of their constituents.

## 2. Experimental

### 2.1. General Experimental Procedure

All the chemicals, reagents, and solvents were analytical/HPLC grade. The essential oil and smoke obtained from the leaves of *A. absinthium* and *H. abyssinica* were analyzed by using an Agilent Technology 7820A GC system coupled with an Agilent Technology 5977E MSD following the procedure described by Sisay et al. [15]. A DB-1701 column (30 × 0.25 *μ*m) was used for chromatographic separation with helium (99.999%) as carrier gas at constant flow rate (0.97989 ml/min). 1 *μ*l of the sample was injected with a splitless mode into the inlet heated to 275°C. The initial column temperature was 60°C and hold time of 2 min with a total run time of 29.33 min. The different components of the essential oil and smoke were identified through NIST 2014 library search and retention index (RI) calculation.

### 2.2. Plant Materials

**Figure 1:**
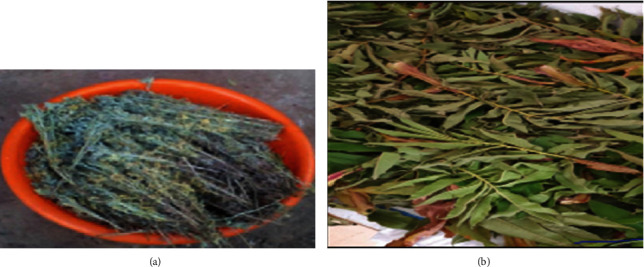
(a) *A. absinthium* and (b) *H. abyssinica* leave sample.

*A. absinthium* (Figure 1(a)) and *H. abyssinica* (Figure 1(b)) leaves were collected from Bati City, Wollo (11°11′N 40°1′E), in Amhara Regional State, eastern part of Ethiopia. The plant specimens were identified by the Department of Biology, Wollo University, Dessie, Ethiopia.

### 2.3. Extraction of Essential Oil from the Leaves of *Artemisia absinthium* and *Hagenia abyssinica* by Hydrodistillation

The essential oil from the leaves of *A. absinthium* and *H. abyssinica* was extracted following the procedure described by Costa et al. [16]. The dried and ground leaves of *A. absinthium* and *H. abyssinica* each (100.0 g) were placed in a distillation flask containing distilled water (500.0 ml). The distillation flask was attached to a Clevenger apparatus and a condenser which was then heated to boiling. The essential oil was separated from aqueous layer and analyzed by GC-MS.

### 2.4. Collection of the Smoke from the Leaves of *Artemisia absinthium* and *Hagenia abyssinica*

The smoke from the leaves of *A. absinthium* and *H. abyssinica* was collected following the procedure described by Sisay et al. [15]. Dried and powdered leaves of *A. absinthium* and *H. abyssinica* each (100.0 g) were burned using an electrical stove, and the smoke was collected using an inverted funnel fitted with a rubber tube which was allowed to pass thorough a suction flask (250.0 ml) containing MeOH (100.0 ml) which in turn was connected to another (250.0 ml) suction flask that contained *n*-hexane (100.0 ml). The side arm of the *n*-hexane-containing flask was attached to a water aspirator. The extracts were dried using anh. Na_2_SO_4_, filtered and concentrated under reduced pressure to yield MeOH (1.50 g) and *n*-hexane (250.15 mg) residues for the leaves of *A. absinthium* and MeOH (1.25 g) and *n*-hexane (150.50 mg) residues for the leaves of *H. abyssinica*, which were then analyzed by GC-MS. Retention index (RI) was calculated for the different components in the essential oil and smoke. For retention index calculation, a mixture of *n*-alkanes (C7–C23) was injected and analyzed using the same experimental condition as that of the essential oil analysis. The retention indices of the different components were then calculated according to the van den Dool and Kratz relationship [17].

## 3. Results and Discussion

### 3.1. GC-MS Characterization of the Essential Oil and Smoke Derived from the Leaves of *Artemisia absinthium*

**Figure 2:**
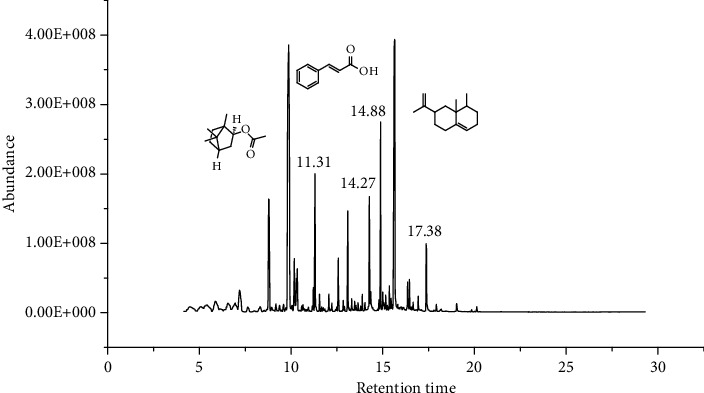
GC-MS chromatogram of *A. absinthium* essential oil.

*A. absinthium* yielded 2 ml of essential oil (1.71 g, 1.71%) and MeOH- (1.50 g) and *n*-hexane- (250.15 mg) trapped smoke residues. *A. absinthium* essential oil showed eighteen components, with different retention times and amount (Figure 2). The main detected components of the oil were valencene (5.48%), bornyl acetate (5.15%), and *trans*-cinnamic acid (4.34%) which together constituted 14.97% of the extracted oil which is comparable with that reported in the literature (Table 1) [4–8].

**Figure 3:**
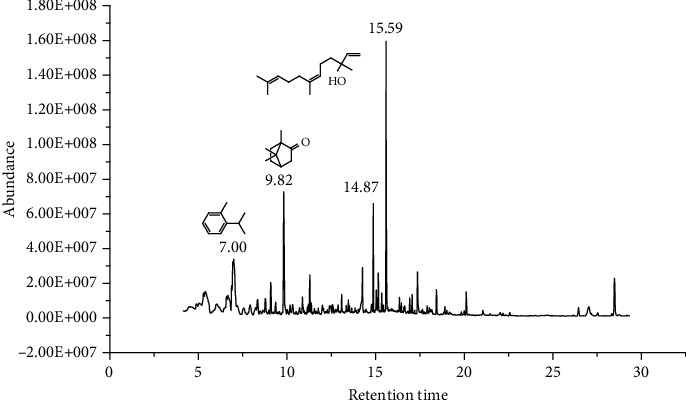
GC-MS chromatogram of *A. absinthium* smoke (MeOH fraction).

**Figure 4:**
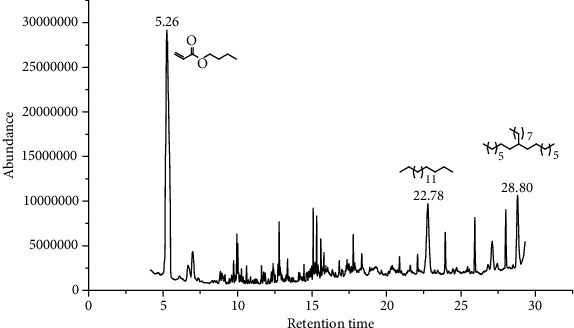
GC-MS chromatogram of *A. absinthium* smoke (*n*-hexane fraction).

27 compounds, 9 from the MeOH-soluble portion and 18 from the *n*-hexane fraction, of the smoke obtained from the leaves of *A. absinthium* were identified (Figures 3 and 4). 2-Bornanone (18.54%), *o*-cymene (12.80%), and nerolidol (7.04%) were the dominant components of MeOH fraction of the smoke derived from the leaves of *A. absinthium* (Table 2). In addition, 2-propenoic acid butyl ester (46.49%), heptadecane (10.66%), and 9-octylheptadecane (7.78%) were the dominant components of *n*-hexane fraction of the smoke derived from the leaves of *A. absinthium* (Table 3). The chemical composition of the smoke obtained from burning the leaves of *A. absinthium* was reported here for the first time.

### 3.2. GC-MS Characterization of the Essential Oil and Smoke Derived from the Leaves of *Hagenia abyssinica*

**Figure 5:**
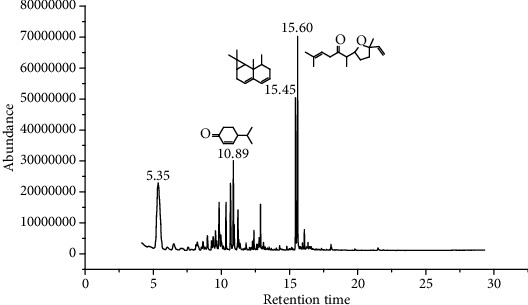
GC-MS chromatogram of *H. abyssinica* essential oil.

1.25 ml of essential oil was collected from the leaves of *H. abyssinica* (1.07 g, 1.07%), and MeOH (1.25 g) and *n*-hexane (150.50 mg) solvent-trapped residues were obtained from the leaves of *H. abyssinica*. The main components of *H. abyssinica* essential oil were *cis*-davanone (14.73%), Aristolediene (9.31%), and cryptone (6.50%); these together constituted 30.54% of the extracted essential oil (Figure 5 and Table 4).

**Figure 6:**
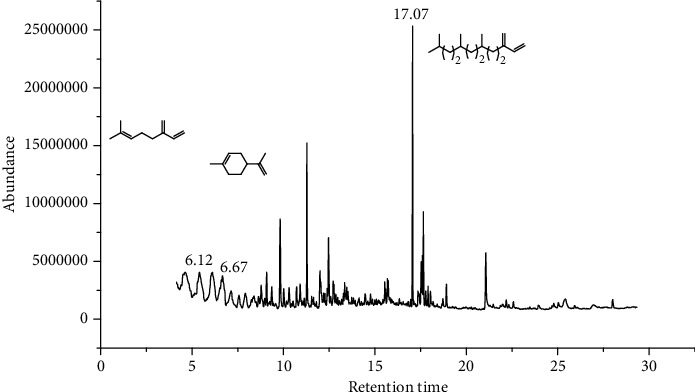
GC-MS chromatogram of *H. abyssinica* smoke (MeOH fraction).

**Figure 7:**
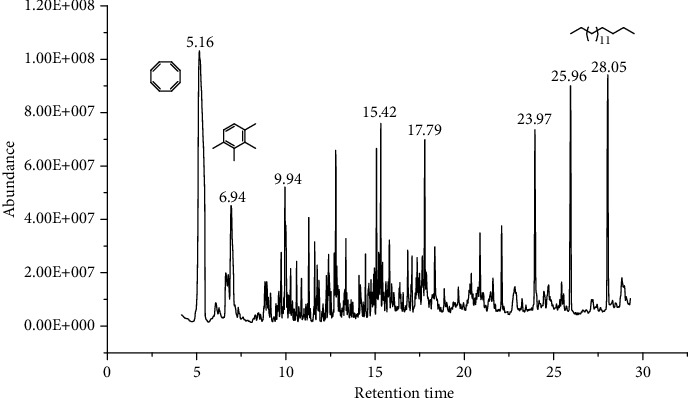
GC-MS chromatogram of *H. abyssinica* smoke (*n*-hexane fraction).

39 compounds, 15 from the MeOH-soluble fraction and 24 from the *n*-hexane fraction, of the smoke obtained from the leaves of *H. abyssinica* were identified (Figures 6 and 7). *β*-Myrcene (12.59%), neophytadiene (11.54%), and limonene (11.27%) were the dominant components of MeOH fraction of the smoke derived from the leaves of *H. abyssinica* (Table 5). 1,3,5,7-Cyclooctatetraene (33.58%), prehnitene (7.01%), and heptadecane (5.46%) were the dominant components of the *n*-hexane fraction of the smoke derived from the leaves of *H. abyssinica* (Table 6). The smoke components of *H. abyssinica* were reported here for the first time.

The essential oil from the leaves of *A. absinthium* yielded a characteristic blue oil which is reported to be due to an artifact product chamazulene [4]. Bornyl acetate, *trans*-ethyl cinnamate, and davanone were found to be the common components of the essential oil from the leaves of *A. absinthium* and the MeOH-soluble fraction obtained by burning the leaves of *A. absinthium*. Oxidation products were also observed in the MeOH-soluble fraction such as 2-bornanone which is the oxidation product of the essential oil component borneol. Davanone was the common component in the essential oil from the leaves of *A. absinthium* as well as the MeOH- and hexane-soluble fractions obtained by burning the leaves of *A. absinthium*. Davanone was previously reported to be the major constituent of the essential oil of the leaves of *A. absinthium* [4]. The chemical constituents of essential oils from the *Artemisia* genus have been extensively studied around the world. Camphor, chamazulene, *β*-myrcene, *β*-pinene, *trans*-sabinyl acetate, and *β*-thujone were reported to be the major constituents of *A. absinthium* different parts from different geographic locations using various extraction methods [18]. The essential oil from the leaves of *H. abyssinica* was found to be dominated by terpenes (30.79%) and aromatic alcohols (8.85%). Terpenes (41.09%) and long chain hydrocarbons (63.04%) were the predominant constituents of the MeOH- and *n*-hexane-soluble fractions obtained by burning the leaves of *H. abyssinica*, respectively. Several previous studies suggested that the main chemical constituents of *H. abyssinica* are phloroglucinol derivatives, phenols, saponins, flavonoids, anthraquinones, terpenoids, alkaloids, steroids, glycosides, and tannins [19].

## 4. Conclusion

In this work, the essential oil from the leaves of *A. absinthium* and *H. abyssinica* together with the smoke obtained by burning the leaves of *A. absinthium* and *H. abyssinica* was analyzed by GC-MS. Eighteen and eleven compounds were identified from the essential oil of *A. absinthium* and *H. abyssinica*, respectively. The smoke from the leaves of *A. absinthium* yielded 27 compounds, 9 from the MeOH-soluble portion and 18 from the *n*-hexane fraction. In addition, 39 compounds, 15 from the MeOH-soluble fraction and 24 from the *n*-hexane fraction, of the smoke obtained from the leaves of *H. abyssinica* were identified.

## Figures and Tables

**Table 1:** Composition of *A. absinthium* essential oil.

PK	RT	Area%	Compound	Structure	Q	RI
1	7.20	2.89	*γ*-Terpinene	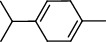	97	1085.94
2	10.18	2.36	Borneol	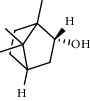	78	1287.17
3	10.30	1.22	2-Ethenyl-6-methyl-5-hepten-1-ol	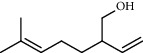	95	1295.03
4	10.34	1.58	*α*-Terpineol	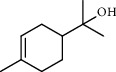	95	1298.37
5	11.22	0.97	Methyl hexa-2,4-dienoate	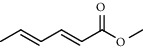	80	1362.33
6	11.31	5.15	Bornyl acetate	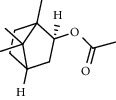	81	1368.81
7	11.56	0.46	Myrcenyl acetate	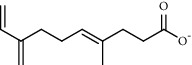	91	1387.18
8	12.58	1.82	Ethyl hydrocinnamate	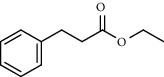	86	1465.43
9	12.85	0.29	*trans*-*β*-Bergamotene	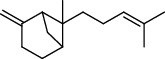	95	1486.44
10	13.10	3.48	*trans*-Ethyl cinnamate	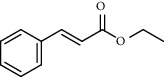	70	1506.13
11	14.27	4.34	*trans*-Cinnamic acid	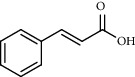	86	1604.47
12	14.88	5.48	Valencene	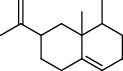	87	1657.58
13	15.01	0.62	Artedouglasia oxide B	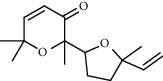	91	1668.31
14	15.17	0.48	Davanone	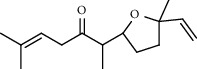	94	1682.17
15	15.46	0.41	Spatulenol	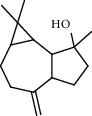	89	1707.82
16	16.36	1.07	Eudesmol	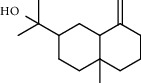	90	1790.78
17	16.94	0.48	Methyl jasmonate	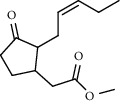	99	1839.13
18	17.38	2.81	1,6-Dimethyl-3-ethylnaphthalene	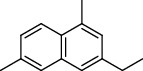	70	1875.20

PK = peak number; RT = retention time; Area% = area percentage; Q = quality; RI = retention index.

**Table 2:** Composition of *A. absinthium* smoke (MeOH fraction).

PK	RT	Area%	Compound	Structure	Q	RI
1	7.00	12.80	*o*-Cymene	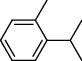	95	1072.87
2	9.09	3.67	Phenol	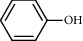	95	1210.85
3	9.82	18.54	2-Bornanone	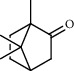	98	1261.99
4	11.29	3.45	Bornyl acetate	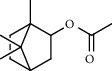	96	1367.57
5	14.26	6.65	*trans*-Ethyl cinnamate	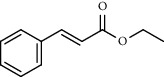	98	1603.39
6	14.87	7.04	Nerolidol	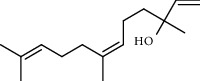	91	1656.50
7	15.16	3.97	Davanone	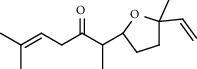	91	1681.44
8	17.37	4.27	Chamazulene	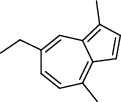	99	1874.48
9	18.44	2.31	Hydroxydavanone	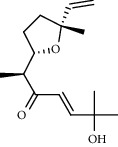	98	1956.30

PK = peak number; RT = retention time; Area% = area percentage; Q = quality; RI = retention index.

**Table 3:** Composition of *A. absinthium* smoke (*n*-hexane fraction).

PK	RT	Area%	IUPAC name	Structure	Q	RI
1	5.26	46.49	2-Propenoic acid, butyl ester	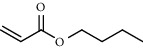	78	960.03
2	7.00	3.66	*p*-Cymene	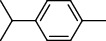	95	1072.65
3	9.96	1.53	2,6,11-Trimethyldodecane	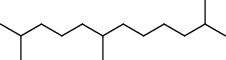	81	1271.47
4	10.02	1.38	Isophorone	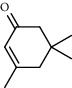	91	1275.75
5	10.61	0.48	Pentadecane	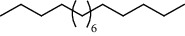	70	1317.59
6	11.62	0.49	Tetradecane	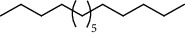	96	1391.66
7	12.39	1.09	2,6,10-Trimethyltridecane	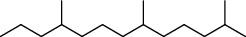	96	1451.15
8	12.80	1.33	Heneicosane	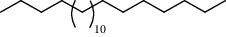	72	1482.84
9	15.09	1.82	2,4-Di-tert-butylphenol	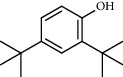	97	1675.11
10	15.60	1.15	*cis*-Davanone	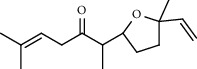	91	1721.26
11	16.83	0.80	9-Methylnonadecane	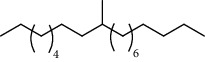	93	1830.59
12	18.35	1.62	Pentacosane	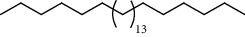	80	1949.41
13	20.88	0.61	Eicosane	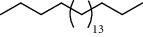	90	2118.98
14	22.78	10.66	Heptadecane	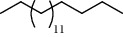	94	2231.49
15	25.94	2.29	Tetracosane	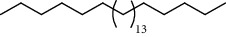	98	2425.72
16	27.10	3.38	Squalene	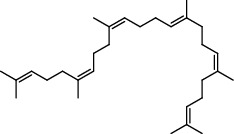	97	2497.57
17	28.02	2.78	Hexacosane	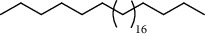	94	2553.80
18	28.80	7.78	9-Octylheptadecane	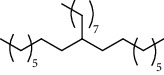	93	2602.25

PK = peak number; RT = retention time; Area% = area percentage; Q = quality; RI = retention index.

**Table 4:** Composition of *H. abyssinica* essential oil.

PK	RT	Area%	Compound	Structure	Q	RI
1	8.98	1.98	Benzyl alcohol	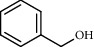	96	1203.47
2	9.84	5.65	2-Bornanone	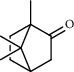	98	1263.10
3	10.35	3.45	2,6,6-Trimethyl-2-cyclohexene-1,4-dione	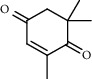	92	1298.51
4	10.89	6.50	Cryptone	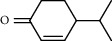	97	1337.86
4	10.95	1.68	*cis*-Carveol	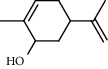	95	1342.96
6	12.40	1.23	*p*-Cymen-7-ol	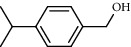	96	1451.49
7	12.78	1.64	2-Methoxy-4-vinylphenol	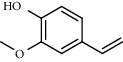	93	1481.30
8	12.88	2.32	*o*-Cymen-5-ol	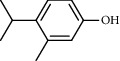	94	1488.86
9	15.45	9.31	Aristolediene	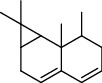	96	1707.62
10	15.60	14.73	*cis*-Davanone	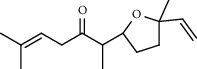	94	1721.10
11	16.10	1.10	Cypera-2,4-diene	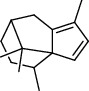	60	1766.60

PK = peak number; RT = retention time; Area% = area percentage; Q = quality; RI = retention index.

**Table 5:** Composition of *H. abyssinica* smoke (MeOH fraction).

PK	RT	Area%	Compound	Structure	Q	RI
1	5.41	8.71	2,5-Dimethylfuran	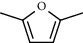	72	969.67
2	6.12	12.59	*β*-Myrcene	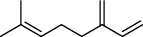	91	1015.81
3	6.67	11.27	Limonene	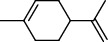	98	1051.59
4	9.09	2.36	Phenol	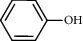	91	1210.78
5	9.82	7.40	Camphor	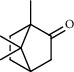	98	1262.10
6	10.72	2.01	2,3-Dihydro-3,5-dihydroxy-6-methyl-4H-pyran-4-one	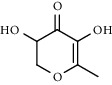	70	1325.92
7	11.29	8.68	Geraniol	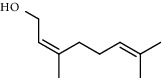	93	1367.28
8	12.47	5.00	1,4:3,6-Dianhydro-alpha-d-glucopyranose	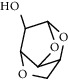	96	1457.20
9	13.35	1.19	Catechol	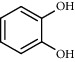	70	1527.54
10	15.55	1.15	1H-Cycloprop[e]azulene	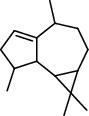	98	1716.28
11	15.69	0.86	1-Methyl-2-pentylcyclopropane	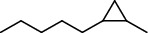	70	1729.27
12	17.07	11.54	Neophytadiene	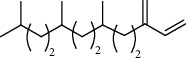	94	1849.48
13	17.54	2.59	D-Allose	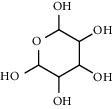	72	1888.31
14	18.91	1.50	Methyl palmitate	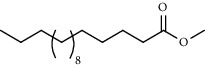	98	1991.63
15	21.07	4.89	Palmitic acid	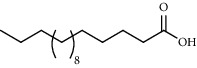	99	2129.94

PK = peak number; RT = retention time; Area% = area percentage; Q = quality; RI = retention index.

**Table 6:** Composition *H. abyssinica* smoke (*n*-hexane fraction).

PK	RT	Area%	Compound	Structure	Q	RI
1	5.16	33.58	1,3,5,7-Cyclooctatetraene	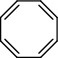	91	953.36
2	6.64	1.89	Limonene	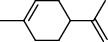	95	1049.69
3	6.94	7.01	Prehnitene	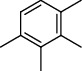	90	1068.88
4	10.00	1.81	Isophorone	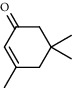	91	1274.68
5	10.27	0.71	Tridecane	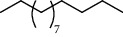	94	1293.10
6	10.61	0.76	2,6,11-Trimethyldodecane	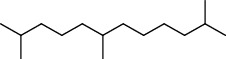	81	1317.42
7	10.88	0.66	4-(1-Methylethyl)-2-cyclohexen-1-one	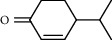	96	1337.14
8	11.29	1.13	*β*-Pinene	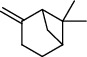	86	1367.49
9	11.62	1.15	Tetradecane	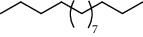	98	1391.51
10	12.27	0.66	Eicosyl vinyl ester carbonic acid	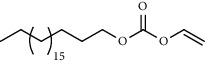	91	1441.78
11	12.39	2.03	Dodecane	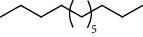	86	1451.12
12	14.47	0.87	Octacosane	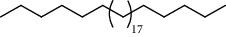	80	1621.29
13	14.98	0.61	Eicosane	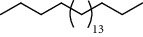	91	1666.17
14	15.09	2.14	2,4-Di-tert-butylphenol	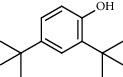	83	1675.17
15	15.42	0.77	9-Octylheptadecane	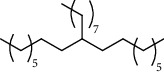	91	1704.36
16	15.81	1.15	2-Methyloctacosane	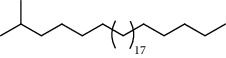	86	1739.94
17	16.84	1.35	10-Methyleicosane	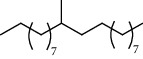	93	1830.79
18	17.07	1.20	Neophytadiene	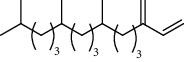	91	1850.18
19	17.36	0.83	Octadecane	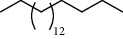	91	1873.92
20	17.65	1.36	Bisabolone	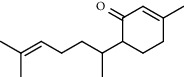	95	1897.42
21	17.79	3.20	Heneicosane	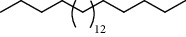	96	1907.68
22	23.97	4.16	Hexadecane	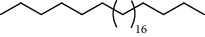	97	2304.26
23	25.96	5.21	Tetracosane	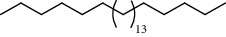	91	2427.25
24	28.05	5.46	Heptadecane	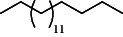	93	2555.63

PK = peak number; RT = retention time; Area% = area percentage; Q = quality; RI = retention index.

## Data Availability

The numerical data used to support the findings of this study are available in the manuscript and can also be obtained from the corresponding author upon request (corresponding author's email: melak.et@gmail.com).
